# Exploration of the Germline Genome of the Ciliate *Chilodonella uncinata* through Single-Cell Omics (Transcriptomics and Genomics)

**DOI:** 10.1128/mBio.01836-17

**Published:** 2018-01-09

**Authors:** Xyrus X. Maurer-Alcalá, Rob Knight, Laura A. Katz

**Affiliations:** a Department of Biological Sciences, Smith College, Northampton, Massachusetts, USA; b Program in Organismic and Evolutionary Biology, University of Massachusetts—Amherst, Amherst, Massachusetts, USA; c Department of Pediatrics, University of California San Diego, San Diego, California, USA; d Department of Computer Science and Engineering, University of California San Diego, San Diego, California, USA; University of California Los Angeles

**Keywords:** Chilodonella, germline, ciliates, genomics, protists, transcriptomics

## Abstract

Separate germline and somatic genomes are found in numerous lineages across the eukaryotic tree of life, often separated into distinct tissues (e.g., in plants, animals, and fungi) or distinct nuclei sharing a common cytoplasm (e.g., in ciliates and some foraminifera). In ciliates, germline-limited (i.e., micronuclear-specific) DNA is eliminated during the development of a new somatic (i.e., macronuclear) genome in a process that is tightly linked to large-scale genome rearrangements, such as deletions and reordering of protein-coding sequences. Most studies of germline genome architecture in ciliates have focused on the model ciliates *Oxytricha trifallax*, *Paramecium tetraurelia*, and *Tetrahymena thermophila*, for which the complete germline genome sequences are known. Outside of these model taxa, only a few dozen germline loci have been characterized from a limited number of cultivable species, which is likely due to difficulties in obtaining sufficient quantities of “purified” germline DNA in these taxa. Combining single-cell transcriptomics and genomics, we have overcome these limitations and provide the first insights into the structure of the germline genome of the ciliate *Chilodonella uncinata*, a member of the understudied class *Phyllopharyngea*. Our analyses reveal the following: (i) large gene families contain a disproportionate number of genes from scrambled germline loci; (ii) germline-soma boundaries in the germline genome are demarcated by substantial shifts in GC content; (iii) single-cell omics techniques provide large-scale quality germline genome data with limited effort, at least for ciliates with extensively fragmented somatic genomes. Our approach provides an efficient means to understand better the evolution of genome rearrangements between germline and soma in ciliates.

## IMPORTANCE

Our understanding of the distinctions between germline and somatic genomes in ciliates has largely relied on studies of a few model genera (e.g., *Oxytricha*, *Paramecium*, *Tetrahymena*). We have used single-cell omics to explore germline-soma distinctions in the ciliate *Chilodonella uncinata*, which likely diverged from the better-studied ciliates ~700 million years ago. The analyses presented here indicate that developmentally regulated genome rearrangements between germline and soma are demarcated by rapid transitions in local GC composition and lead to diversification of protein families. The approaches used here provide the basis for future work aimed at discerning the evolutionary impacts of germline-soma distinctions among diverse ciliates.

## INTRODUCTION

For most “textbook” eukaryotes, the genome is often viewed as identical in every cell. However, any organism with established germline and somatic cells harbors numerous distinct genomes in part due to the potential differences in ploidy (e.g., N in germline-nuclei compared to 2N in somatic tissues for plants and animals). Differences between germline and soma extend beyond ploidy, with numerous studies documenting the developmental genome rearrangements (e.g., changes in genome architecture) that occur during cellular differentiation into specific tissues, such as the V(D)J recombination in the immune system of vertebrates (1, 2). Additional examples of developmentally regulated genome rearrangements include formation of extrachromosomal ribosomal DNAs and antigen switching in parasites, and such processes are found throughout the eukaryotic tree of life (3–9).

In ciliates, a clade of microbial eukaryotes that is estimated to be about 1 billion years old (10), germline and somatic functions are isolated into distinct nuclei within a single cell/individual. As in animals, the germline remains quiescent throughout much of a ciliate’s life, only becoming transcriptionally active during conjugation (i.e., sex in ciliates). In *Chilodonella uncinata* (in the class *Phyllopharyngea*), the germline genome is composed of more “traditional” chromosomes (11–13) while the somatic chromosomes are present as “gene-sized” nanochromosomes that are maintained at variable copy numbers. As a result, this ciliate, described as having an extensively fragmented genome, has a somatic nucleus that harbors >20 million nanochromsomes (14–16).

Because of difficulties in culturing and the high level of amplification of somatic genomes compared to the germline (which contributes to contamination in germline DNA preps), traditional methods for sequencing germline-limited DNA are fairly laborious and costly in terms of time and benchwork. This has led to limitations in the phylogenetic breadth of explorations of ciliate germline genomes to a few model species for which cultures can provide sufficient numbers of cells (often in the millions) and for which time-tested germline isolation techniques exist. The limitations on the ability to extract quality germline micronuclear DNA with sufficient yields for high-throughput library construction, especially considering the loss of DNA associated with each manipulation and purification step (17), has likely been the greatest barrier to studies of germline genomes in non-model ciliates.

The emergence of single-cell omics techniques enables us to employ single-cell genomics and transcriptomics for the first large-scale exploration of germline genome architecture in the extensively fragmenting ciliate *Chilodonella uncinata* in the class *Phyllopharyngea*. By taking advantage of the biochemical bias in multiple displacement amplification toward large chromosomes (i.e., long template DNA) during whole-genome amplification reactions (18–20), we have been able to assemble and explore a substantial portion of the germline genome of *C. uncinata*.

In this study, we demonstrate the power of single-cell omics to provide insights into germline genomes in ciliates with gene-sized chromosomes. In addition to providing a summary of general features of the *C. uncinata* germline genome architecture, we have used the data generated here and those data for other ciliate species to show how dramatic shifts in local GC content distinguish somatically destined DNA from germline-limited DNA. We also describe how the germline genome architecture is associated with gene family size; in *C. uncinata*, the largest gene families, which appear *Chilodonella* specific, are enriched with scrambled genes. This supports the model showing that scrambling and alternative processing are ways that ciliates are able to increase protein diversity (12, 13).

## RESULTS

### Recovery of germline sequences from single-cell omics techniques.

To explore the germline genome architecture of *Chilodonella uncinata*, we compared the characterization of germline sequences derived from a “traditional” gel-based method to those from a single-cell omics approach. Our traditional method requires the cultivation of large numbers of cells, total DNA isolation, enrichment for large germline chromosomes, and treatment with Bal31 to remove somatic contaminants; the last step of this process is difficult to optimize, given the time required to obtain a sufficient number of cells (~2 to 3 weeks). In contrast, the single-cell omics approach relies on the Qiagen REPLI-g single-cell kit to amplify the germline genome; with this approach, the reliance on the high-fidelity Phi-29 polymerase provides selectivity for larger germline chromosomes over short somatic chromosomes (see Materials and Methods). Our pilot assessment of the traditional DNA isolation and single-cell approaches revealed substantially more “somatic” contamination in the traditional approach, measured by the number of assembled scaffolds that were bounded by 1 or more telomeres (>2 orders of magnitude) (see Table S1 in the supplemental material). Similarly, we were able to identify a far greater number of putative germline loci by using the single-cell approach rather than the traditional approach (>5,000 loci versus ~400 loci) (Table S1). Given these data, we proceeded to further analyze only the single-cell omics-derived data.

10.1128/mBio.01836-17.2 TABLE S1  Comparisons of germline genome assemblies based on the germline DNA gel isolation method and single-cell techniques, demonstrating the superiority of single-cell WGA (putative germline scaffolds are those with predicted ORFs across <20% of their length, while supported germline scaffolds have at least 3 transcripts that align to the scaffold; somatic contamination, e.g., presence of telomere-containing scaffolds, are far more common and problematic in assemblies of gel-isolated germline DNA; similarly, use of BLAST to map transcripts to the independent assemblies further demonstrates the superiority of the single-cell approach). Download TABLE S1, DOCX file, 0.01 MB. Copyright © 2018 Maurer-Alcalá et al. 2018 Maurer-Alcalá et al. This content is distributed under the terms of the Creative Commons Attribution 4.0 International license.

### Patterns of genome rearrangements inferred from germline sequences.

To assess the resulting germline sequences from single-cell 'omics, we mapped transcripts, which are a proxy for the gene-sized macronuclear chromosomes of *Chilodonella uncinata*, to putative micronuclear scaffolds generated using the single-cell omics approach. Using our requirement of ≥60% coverage for each transcript, we mapped 5,019 transcripts (~40% of the total assembled *C. uncinata* transcriptome) to over 32.7 Mbp of the germline genome. A total of 7,448 transcripts remained unmapped to the germline assembly, indicating that additional sequencing efforts are required to completely sequence the germline genome. Nevertheless, we estimated the size of the germline genome based on gene number to be ~22,500 from the somatic genomes of *Oxytricha* (21) and *Stylonychia* (22) (ciliates that also have extensively fragmented somatic genomes and are distantly related to *C. uncinata*). Using a range for overall gene content (~15,000 to 22,500 genes) and our ability to map ~5,000 transcripts across ~33 Mbp (~150 genes per Mbp), we estimated a germline genome size of ~99 to 149 Mbp for *Chilodonella uncinata*. This estimate will be refined with additional sequencing, as we expect variation among ciliates in the proportion of repetitive regions (e.g., microsatellites, transposons, and centromeres).

**FIG 1:**
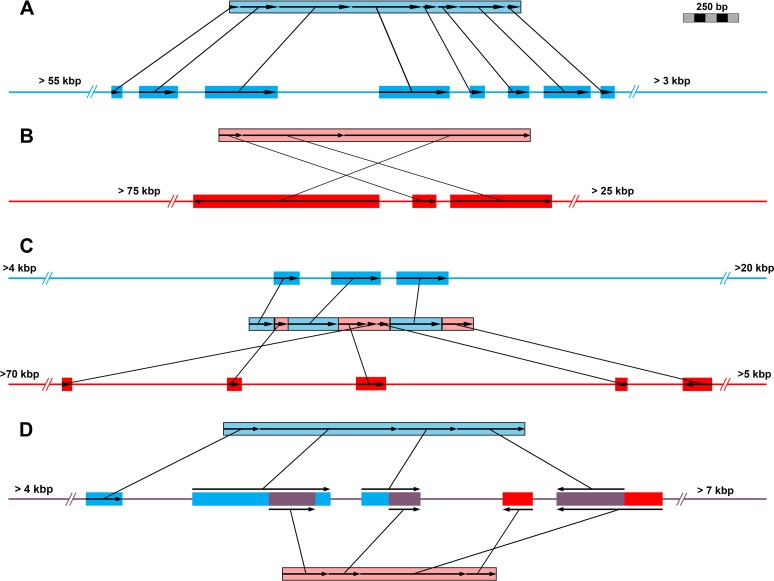
Exemplar patterns of genome architecture from the germline-mapped transcriptome data of *Chilodonella uncinata*. Germline loci are represented as a single line harboring MDSs (colored rectangles). (A) Typical nonscrambled germline genome architecture. (B) Exemplar scrambled germline locus. (C) Processing of two distant germline loci into single somatic sequence. (D) Alternative processing of a single germline locus produces two distinct somatic sequences. Arrows indicate directionality of macronuclear destined sequences.

Mapping transcripts allowed us to identify the proportion of genes from nonscrambled versus scrambled germline loci. Nonscrambled loci are those whose transcripts map to macronuclear destined sequences (MDSs) maintained in consecutive order and those lacking evidence of internally eliminated sequences (IESs, i.e., germline-limited DNA) (Fig. 1A). We identified scrambled loci as those meeting two criteria: (i) existence of MDS-IES boundaries with identifiable pointer sequences (i.e., short direct repeats required for unscrambling) and (ii) MDSs in a nonconsecutive order and/or MDSs found on both strands of the germline scaffolds (i.e., some are inverted) (Fig. 1B to D). Of these mapped transcripts, we found 3,475 (69%) cases of nonscrambled loci in the germline (Fig. 1A; Table 1), while 1,544 (31%) loci showed strong evidence of scrambling (including alternative processing of germline loci) (Fig. 1B to D; Table 1).

**TABLE 1:** Nonscrambled and scrambled germline loci differ substantially in numerous basic features^a^

Feature	Scrambled	Nonscrambled
No. of mapped transcripts	1,544	3,475
MDS no.	3.29* (4)	2.46* (2)
MDS length (bp)	160.96* (133)	212.20* (179)
Pointer length (bp)	8.59* (8)	6.55* (6)
% GC content of MDS-IES	41.25 (41.09)	39.61 (39.80)
Distance between pointers (bp)	1,454.89* (805)	136.78* (104)

^a^ All values in parentheses represent median values for a given category. *, significant difference between scrambled and germline loci (*P* < 0.05).

Scrambled and nonscrambled germline loci differed in several key features (Table 1). Scrambled genes tend to be more fragmented in the germline—composed of a greater number of MDSs—than nonscrambled transcripts (3.29 and 2.46, respectively; *P* << 0.05). Moreover, these MDSs are also significantly shorter in length than nonscrambled loci (161.0 bp versus 212.2 bp, respectively; *P* << 0.05). Similarly, scrambled gene loci tend to have longer pointers (8.59 bp versus 6.55 bp, respectively; *P* << 0.05). We found that the consecutive MDSs of scrambled germline loci (found on the same germline scaffold) were separated by far greater distances than their nonscrambled counterparts (1,454.89 bp versus 136.78 bp, respectively; *P* << 0.05).

### GC composition at MDS-IES boundaries.

**FIG 2:**
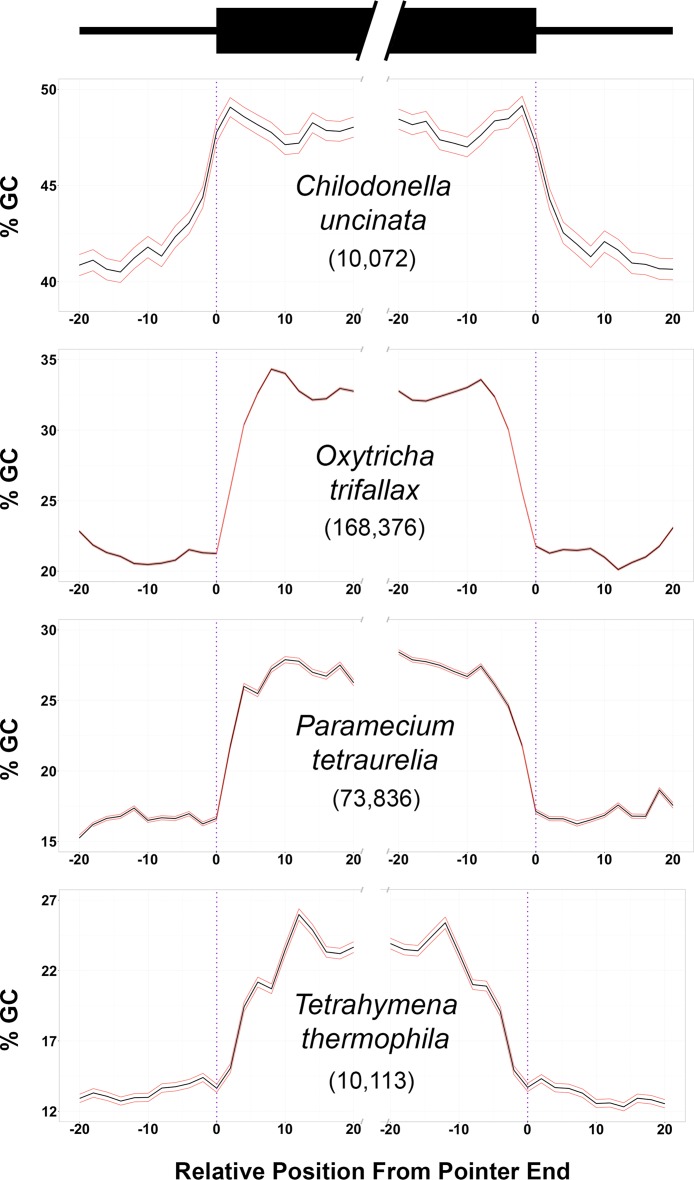
Sharp increases in local GC content are associated with germline-soma boundaries in diverse ciliates. The sliding window average (3 bp; black) of GC content with 95% confidence intervals (red) are shown. Values under taxon names indicate the number of MDS-IES boundaries examined. Data for *C. uncinata* are from this study, and data from other ciliates are from GenBank (see Materials and Methods).

We examined the distribution of GC content on both small scales, focusing on identifiable MDS-IES boundaries, and broad scales, to assess fluctuations across entire assembled scaffolds. Average GC content at MDS-IES boundaries in *C. uncinata* did not differ between scrambled and nonscrambled MDSs (41.25% and 39.61%, respectively; *P* > 0.05) (Table 1), and so we combined these data for further comparisons. By focusing on a 40-bp window on both the 5′ and 3′ ends of MDSs, we observed a substantial change in GC composition (~12% difference) at MDS-IES boundaries in *C. uncinata*, with greater GC content in MDSs than in the neighboring micronuclear-limited sequences (Fig. 2).

We also looked at this small-scale relationship in the few other ciliates either with complete germline genomes (e.g., *Oxytricha trifallax* and *Tetrahymena thermophila*) or with thousands of inferred MDS-IES boundaries (e.g., *Paramecium tetraurelia*) (Fig. 2). Despite relatively large differences in overall GC content in the germline genome data among these divergent taxa (e.g., ~20.67% in *Tetrahymena* and ~49.44% in *Chilodonella*), the boundaries between germline-limited and somatic-destined DNA were marked by sharp changes in GC content (~10 to 14%).

Making use of the observable rapid changes in GC content between germline and somatic regions across broader scales allowed identification of coding domains that did not map to our transcript libraries. Given that sharp transitions in base composition likely delineate MDSs from neighboring germline-limited regions among diverse ciliate taxa, we identified regions (>40 bp) in the *C. uncinata* germline scaffolds that had significantly greater or lower GC contents (>2 standard deviations) compared to the average GC content of the assembly. We used BLAST to determine if these regions with extreme composition bias had homologues in other organisms. Of the 250 largest regions with atypically high GC content (average, 1,065 bp), 136 regions (54.4%) had significant BLAST hits (E values of <1e^−10^) with other eukaryotes, predominantly ciliates, whereas only 1 of the 250 largest regions (<1%; average, 580 bp) with significantly lower GC content had a homologue to another organism (Table S2); the functional significance (if any) of regions with very low GC content remain to be discovered.

10.1128/mBio.01836-17.3 TABLE S2  Top BLAST hits for the largest 250 regions of germline scaffolds without mapped transcriptome data with a GC content that is significantly above or below the average GC content (the majority of these atypically GC-rich regions from the *C. uncinata* germline genome had homologues in other eukaryote taxa, predominantly other ciliate taxa and *Alveolates*). Download TABLE S2, DOCX file, 0.01 MB. Copyright © 2018 Maurer-Alcalá et al. 2018 Maurer-Alcalá et al. This content is distributed under the terms of the Creative Commons Attribution 4.0 International license.

### Gene scrambling and gene family size evolution.

**FIG 3:**
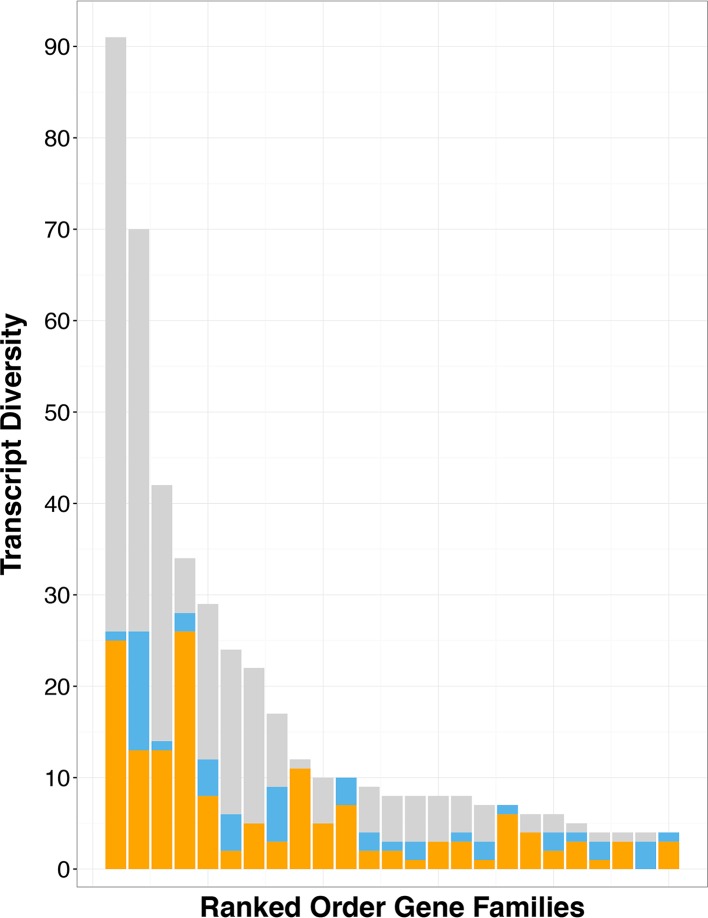
*Chilodonella uncinata*’s largest (most diverse) gene families are composed of scrambled genes. Contributions to gene family size by scrambled genes (orange) are typically far greater than those of nonscrambled genes (blue), despite the large number of unmapped transcripts (gray). The proportion of scrambled transcripts in each of these large families was significantly greater than expected (*P* << 0.05) given their overall abundance.

To assess the impact of gene scrambling on gene family size, we classified the transcriptome data from *C. uncinata* into gene families by using OrthoMCL’s clustering algorithms (23). We used the number of unique transcripts within a given gene family (referred to as transcript diversity) as an approximation of gene family size, given the potential for partial open reading frames (ORFs) in the transcriptomic data. When we considered only mapped transcripts, the gene families with the greatest observed transcript diversity were disproportionately composed of transcripts with strong signatures of scrambling (Fig. 3). Gene families containing scrambled transcripts were also disproportionately larger (often double in size) than other gene families, with ~2.93 members in scrambled gene families compared to ~1.29 members in nonscrambled gene families (*P* << 0.05). Using the observed overall frequencies of scrambled and nonscrambled transcripts (31% and 69%, respectively) to generate expected proportions of scrambling, we determined that the largest gene families were significantly more often enriched with scrambled gene family members than expected (*P* << 0.05).

## DISCUSSION

In this study, we used single-cell omics to compare the germline and somatic genomes of the ciliate *C. uncinata*, and we demonstrated that (i) germline genome architecture and subsequent processing (e.g., DNA elimination, unscrambling) impact gene family sizes and patterns of molecular evolution in the somatic genome, (ii) substantial shifts in composition (i.e., GC content) in the germline micronucleus demarcate boundaries between somatic coding sequences and germline-limited DNA, and (iii) the use of single-cell molecular approaches provides a robust preliminary look at the germline genome of ciliates with extensively fragmented somatic genomes.

### Feasibility and use of single-cell omics for germline genomes.

In this study, we demonstrated that single-cell omics efficiently provides quality insights into the germline genome architecture of *Chilodonella uncinata*. Currently, the majority of data on germline genome rearrangements and architecture in ciliates is limited to three model ciliates: *Oxytricha trifallax* (24), *Paramecium tetraurelia* (25), and *Tetrahymena thermophila* (26). Yet, these well-studied taxa come from only 2 of the 11 ciliate classes (*Spirotrichea* and *Oligohymenophorea*). Reasons for this limitation include the inability to gather enough starting material for high-throughput sequencing efforts, as well as potential bioinformatic bottlenecks (e.g., assembly-related issues, such as low sequencing coverage). Our combination of single-cell genomic (from four individual cells) and transcriptomic amplification outperformed traditional germline DNA isolation in terms of the number of identifiable germline loci and exploration of general germline features (Table S1). Similarly, the gel isolation-based approach for enrichment of micronuclear DNA is also considerably time inefficient, requiring robust and dense cultures (which may be difficult to generate for some lineages), whereas the single-cell approaches used in this study can be performed within several days and require very few cells and relatively low effort for robust results. Hence, single-cell omics methods provide the means to move beyond the confines of the bench and explore the overall complexity and impacts of genome architectures in uncultivable ciliates and perhaps other microbial eukaryotes.

### Impact of germline genome architecture on evolutionary patterns.

Genome architecture and processing (e.g., DNA elimination, genome rearrangements, and amplification during generation of somatic chromosomes) appear to play roles in gene family evolution in ciliates. F. Gao et al. (12) hypothesized that the patterns of gene family evolution in ciliates (which comprise a few unique families with large numbers of members) may be a consequence of genome processing, which is further supported by our analyses of *C. uncinata*’s germline genome. We found that gene families with the greatest transcript diversity are enriched for genes scrambled in the germline. Scrambled genome architectures are likely to arise through the duplication of germline loci, followed by their partial degradation (11–13). Gene duplication is a driving factor in the expansion of gene families in *C. uncinata* and provides a basis for alternative processing of multiple germline loci (a DNA-based process analogous to alternative exon splicing of mRNAs) (Fig. 3C and D) (11–13). However, it is difficult to distinguish which mechanism, alternative processing or rampant duplication, is the major driver for the observed gene family expansions, given our incomplete germline genome data. Intriguingly, the largest gene families in *C. uncinata* are rich with scrambled transcripts that are expressed during conjugation, as estimated by single-cell transcriptomics (Table S4). These large gene families also appear *Chilodonella* specific, as they lack homologues in other eukaryotes, suggesting that gene duplication and alternative processing contribute to lineage-specific features.

10.1128/mBio.01836-17.4 TABLE S3  Distribution of gene family members based on OrthoMCL clustering (transcript names in a given row indicate transcripts that are members of the same gene family). Download TABLE S3, XLS file, 1 MB. Copyright © 2018 Maurer-Alcalá et al. 2018 Maurer-Alcalá et al. This content is distributed under the terms of the Creative Commons Attribution 4.0 International license.

10.1128/mBio.01836-17.5 TABLE S4  The largest gene families in *C. uncinata* are disproportionately composed of transcripts found during conjugation. Download TABLE S4, TXT file, 0.1 MB. Copyright © 2018 Maurer-Alcalá et al. 2018 Maurer-Alcalá et al. This content is distributed under the terms of the Creative Commons Attribution 4.0 International license.

Compared to other eukaryotic lineages, ciliate genomes tend to be composed of fewer but larger gene families (e.g., gene families with >15 members). For example, the model ciliate *Tetrahymena thermophila*’s somatic genome contains 26,992 protein-coding genes that comprise 8,826 gene families (3.04 members per family), as estimated from OrthoMCL’s gene family clustering. In contrast, other eukaryotes tend to have many more gene families with fewer members. For example, the estimate for *Drosophila melanogaster* is that its 14,422 protein-coding genes fall within 12,925 gene families (1.11 members per family) (27), and for *Arabidopsis thaliana* an estimated 25,498 genes fall into 11,601 different gene families (2.31 members per family) (28). In *C. uncinata*, estimates of gene family sizes based on our transcriptomic data are consistent with data from *T. thermophila*, with *C. uncinata*’s 12,467 transcripts comprising 4,153 families (3.00 transcripts per family). While this may be an overestimate for gene family sizes (given the incomplete nature of transcriptomic data), the lack of major differences in gene family sizes between *T. thermophila* and *Chilodonella* is fairly striking, as our data demonstrate a close relationship between scrambled germline loci and gene family size. The observed bias in the expansion of *Chilodonella*-specific gene families (through gene scrambling) may account for the above estimates, as the sizes of these expanded lineage-specific gene families would not be included. This may be common among ciliates with highly scrambled germline genomes, although this may depend on the number of evolutionary origins of gene scrambling, which remains uncertain.

Although ciliates in both the classes *Phyllopharyngea* (e.g., *C. uncinata*) and *Spirotrichea* (e.g., *O. trifallax*, *S. lemnae*) harbor scrambled loci, the large-scale arrangements of MDSs in their germline genomes differ. While nonscrambled and scrambled genes are often found interdigitated in germline loci in both *O. trifallax* (24) and *C. uncinata*, the somatically destined DNA in the *O. trifallax* germline genome tends to be present in far more tightly compact genomic “islands” (24); the degree of proximity is so close that the typical distance between neighboring MDSs is nearly nonexistent. From our observations, this is not the case for *C. uncinata*, as distances between neighboring MDSs are often relatively large (often >1 kbp apart) (Table 1). This difference is consistent with the proposed independent origins of germline genome scrambling in these divergent taxa (29).

### Compositional bias demarcates germline-soma boundaries.

We demonstrated that MDS-IES boundaries are delineated by rapid shifts in GC content, with germline-limited DNA being GC poor compared to somatic-destined sequences (Fig. 2; Table 1). Using biases in GC content as a tool to understand germline genome architecture, we found visual evidence for well-known differences in the developmental process (e.g., precision of DNA elimination) among ciliates (Fig. 2). For example, almost all IES excision in *T. thermophila* is known to be imprecise and is marked by the greater variability in GC contents associated with MDS-IES boundaries within the inferred MDS itself (~10 bp from the inferred MDS-IES boundary) (Fig. 2). However, in *Paramecium tetraurelia*, which undergoes precise IES excision during development, we observed the opposite: there was a substantial decrease in GC content in much closer proximity to its MDS-IES boundaries (Fig. 2).

The role of compositional bias in marking important genomic features has been well described in model plants and animals, with major transitions in GC richness associated with transcriptional start sites (30, 31) and recombination hot spots (32). As somatic chromosomes in ciliates are far more streamlined (e.g., smaller intergenic regions, lacking centromeres, and intron-poorer genes) (21, 22, 33–35), selection may maintain the strong clines in GC content associated with MDS-IES boundaries as a means of identifying transcriptionally active sequences (soma) within potentially large regions of non-protein-coding DNA (germline-limited DNA). These observations from highly processed ciliate chromosomes are consistent with data from diverse eukaryotes, where GC content in coding domains differs substantially from neighboring intergenic regions (36–40), implicating the role of shifts in GC content as a means for demarcating coding domains despite major differences in genome architecture (e.g., single-gene nanochromosomes versus traditional “long” multigene chromosomes).

## MATERIALS AND METHODS

### Ciliate culturing and DNA extraction.

A clonal line of *Chilodonella uncinata* (Pol strain; ATCC PRA-257) was cultured in filtered and autoclaved pond water at room temperature and in the dark, with sterilized rice grain to support bacterial growth, following published protocols (12, 41, 42). Following traditional protocols, micronuclear-enriched DNA extraction started with ~400,000 cells and relied on gel isolation of high-molecular-weight molecules, as described elsewhere (11–13). Briefly, after purification of DNA from the agarose gel, the enriched high-molecular-weight DNA are digested with Bal 31 for up to 5 min, yielding more greatly micronuclear-enriched DNA that was used for further analyses. Bal 31 is an enzyme that digests double-stranded DNA at a rate of ~100 bp per min per end (43). Given the time required in generating a sufficient number of cells, the 5-min Bal 31 incubation, which equates to ~2 kbp of degraded DNA, was our best guess for sufficient somatic macronucleus (MAC) degradation with limited diploid micronucleus (MIC) destruction. Given the time involved in culturing sufficient numbers of cells, there are no data on the impact of varied times of Bal 31 incubation on MAC contamination.

### Single-cell whole-genome amplification.

For single-cell genomics protocols, we selected vegetative cells (i.e., those not undergoing conjugation or division) from a rapidly growing population. Each cell was washed 5 times in 0.2-µm-filtered pond water to dilute any bacteria that may have been carried over. For whole-genome amplification (WGA), we placed each cell in an individual sterile 0.2-ml tube and followed the Repli-g single-cell kit manufacturer’s instructions (catalog number 150343; Qiagen).

### PCR-based confirmation of whole-genome amplification.

We took advantage of the inherent template length bias of the WGA reaction, which better amplifies “long” (<2-kbp) template DNA (according to the manufacturer) to selectively amplify the long chromosomes of the germline genome. To confirm these results, we used PCR primers designed to specifically amplify macronuclear or scrambled micronuclear forms of actin (based on data from L. A. Katz and A. M. Kovner [13]) (Table S5) for all the WGA products. All WGA products sequenced demonstrated substantial enrichment of the micronuclear arrangement of actin, with no observable amplification of when macronuclear-specific actin primers were used, demonstrating the preference of the WGA reaction for germline DNA templates. In contrast, PCR of the traditional DNA isolation (following Bal 31 treatment) clearly amplified the micronuclear arrangement of actin; there was also evidence for the amplification of the somatic arrangement of actin, although the amplification was far less robust than untreated DNA preparations (i.e., prior to Bal31 treatment).

10.1128/mBio.01836-17.6 TABLE S5  PCR primers used to discriminate between macro- and micronuclear copies of actin. Download TABLE S5, DOCX file, 0.01 MB. Copyright © 2018 Maurer-Alcalá et al. 2018 Maurer-Alcalá et al. This content is distributed under the terms of the Creative Commons Attribution 4.0 International license.

### Single-cell whole-transcriptome amplification.

For whole-transcriptome amplification (WTA), we followed the same cleaning protocol described above but also selected individual cells undergoing division (amitosis), conjugation (sex), and feeding (e.g., vegetatively growing cells) within the clonal cultures to assess major variations in transcription. After washing, the WTA reactions were carried out following the manufacturer’s protocols (Smart-Seq v4 ultralow input RNA kit; Clontech), though we used only one of the four reaction mixtures. Overall, we prepared single-cell transcriptomes for three dividing cells, five active feeding cells, and four pairs of conjugating cells (as we did not want to separate conjugating pairs). These WTAs, representing three major life cycle stages, were used in our analyses.

### Genome and transcriptome sequencing.

We sequenced three types of material: (i) micronuclear-enriched DNA isolated by gel electrophoresis, (ii) WGAs from four individual cells to capture micronuclear DNA, and (iii) 12 WTA from single cells (five vegetative, three dividing, four in conjugation). The micronuclear-enriched DNA, from gel isolation, was sequenced on a single channel on an Illumina HiSeq2500 at the Yale Center for Genome Analysis. The four individual WGAs were later sequenced on a single channel of an Illumina HiSeq4000 at the Genome Resource Center at the University of Maryland at Baltimore. Libraries of the WTAs were constructed using the NexteraXT kit, following the manufacturer’s instructions (Illumina) and then sequenced at the IGM Genome Center at the University of California at San Diego on a portion of a single channel of a HiSeq4000 apparatus. A description of our raw data can be found in Table S1.

### Genome and transcriptome assembly.

Raw reads for both genomes and transcriptome assemblies were assessed and trimmed using BBTools (http://sourceforge.net/projects/bbmap) (44) with a minimum quality score of 28 and minimum length of 125 bp. Following quality trimming, genome data for all four individuals were pooled and assembled using SPAdes (v3.5.0) (45) and MaSuRCA (46). As the continuity of the SPAdes assembly was greater than that of the MaSuRCA assembly (determined as the number of transcripts mapped to the assembly per kilobase), we used the SPAdes assembly for all data analyses reported here. Each single-cell transcriptome was assembled independently using rnaSPAdes (v0.1.1), due to the likely heterogeneity in exact timing for each life stage.

### Preparation of single-cell transcriptome data.

Each of the assembled transcriptomes was processed through a series of custom python scripts, which included updating the name of the transcript to include its representative life cycle stage (e.g., conjugation) and the removal of contaminating rRNA and bacterial transcripts (http://github.com/maurerax/KatzLab/tree/HTS-Processing-PhyloGenPipeline). We then pooled these transcriptomes to remove transcripts of near identity (e.g., >98% identical) across ≥75% of their length to larger transcripts. This reduced pool was considered the “core” *C. uncinata* transcriptome that we used for subsequent analyses.

### Identification of putative germline loci.

To identify germline genome regions, we mapped the prepared core transcriptome (a proxy for macronuclear gene-sized chromosomes) to the long contigs generated from both the gel-isolated high-molecular-weight DNA (from a culture) and the assembled pool of the four single-cell WGAs. To distinguish putative germline loci from bacterial contaminants in the WGA assemblies, we used Augustus (v3.2.1) (47) to predict open reading frames under the available *Escherichia coli* K-12 model. Due to the expected complexity in the germline genome architecture of *C. uncinata* (i.e., ORFs tend to contain internally eliminated sequences demarcated by variable pointer sequences, and some ORFs are scrambled), complete ORFs should be difficult to identify. For characterization of ciliate germline scaffolds, we considered both lower numbers of ORFs as well as higher numbers of matches to the core *C. uncinata* transcriptome: scaffolds of ≥10 kbp with few predicted ORFs and numerous (>3) mapped transcripts were considered putative germline loci and used for further analyses.

### Identification of MDS structure.

After identifying a set of putative *C. uncinata* germline (micronuclear) scaffolds, we used BLAST (v2.4.0) (48) with the parameters -ungapped -perc_identity 97 -outfmt 6 to map transcriptome data along germline scaffolds. Custom python scripts (http://github.com/maurerax/KatzLab/tree/SingleCellGermSoma) were used to analyze the output from BLAST and to categorize the loci and transcriptome data into three broad categories: nonscrambled, scrambled, and unmapped. A range from 30 to 90% of mapped transcript length was explored (Fig. S1), with a greater percentage of mapped values biased against scrambled gene data, among which 60% of mapped values provided the clearest evidence for germline genome architectures. Therefore, only transcripts with ≥60% of their length mapped to the germline assembly were used for subsequent analyses.

10.1128/mBio.01836-17.1 FIG S1  Histograms of mapped transcripts based on the proportions of their lengths mapping to germline scaffolds. These distributions and our confidence in assessments of the genomic architecture of germline loci were used to determine the balance between the number of transcripts mapped and their “realness.” Download FIG S1, TIF file, 0.4 MB. Copyright © 2018 Maurer-Alcalá et al. 2018 Maurer-Alcalá et al. This content is distributed under the terms of the Creative Commons Attribution 4.0 International license.

To ensure that the single-cell assembly was not generating chimeric scaffolds, we checked read coverage maps for multiple genomic scaffolds associated with different germline architectures (scrambled and nonscrambled). We found no evidence to suggest our assemblies were chimeric (e.g., germline-limited DNA between pointer sequences with abnormally low coverage), and we used this assembly for further analyses. To ensure that potential MDS-IES boundaries were not intron-exon boundaries (considering our use of transcripts as a proxy for the somatic genome), in order to characterize a transcript as harboring an IES the IES had to be flanked by identical pointer sequences and not be nearly identical to the canonical GT-YAG intron-exon boundaries.

### Analyses of composition at germline-soma boundaries.

To assess GC composition at MDS-IES boundaries, we used the most recent versions of *Tetrahymena thermophila* and *Oxytricha trifallax*’s macronuclear and micronuclear genomes (micronuclear germline assemblies for *Tetrahymena* and *Oxytricha* are available from GenBank under accession numbers AAGF00000000 and ARYC00000000, with their corresponding macronuclear assemblies, AAGF00000000 and AMCR00000000, respectively). Germline data for *Paramecium tetraurelia* was downloaded from http://paramecium.cgm.cnrs-gif.fr/. For *Tetrahymena* and *Oxytricha*, telomere sequences were removed, and whole macronuclear chromosomes were mapped to their respective germline genome assemblies by using BLAST, as described above. For *Chilodonella*, we used the BLAST report for confirmed germline loci. For *Paramecium*, transitions from MDSs to germline-limited sequences in the available assembly were marked by the shift from uppercase to lowercase characters, which we processed into genomic scaffold coordinates. With the coordinates for these transitions from soma to germline for each taxon, custom python scripts were then used to assess local changes in average GC composition over a sliding 3-bp window with a 2-bp step at MDS-IES boundaries.

### Identification of somatic contamination from germline genome assemblies.

For identification (and removal) of somatic chromosomes from our germline genome assemblies, we removed all scaffolds capped with *Chilodonella*’s telomeric repeat CCCCAAA (35). Specifically, any scaffold with CCCCAAACCCC or AAACCCCAAA found within its first and/or last 30 bp (allowing for a single mismatch) was characterized as somatic and isolated prior to our analyses of the germline genome architecture, which were conducted using custom python scripts. These data are summarized in Table S1.

### Comparison of germline DNA isolation methods.

To compare traditionally isolated germline DNA (i.e., isolated from cultured cells by gel electrophoresis and treatment with Bal 31 nuclease, following protocols reported elsewhere [11–13]) to single-cell genome amplification, we evaluated the putative germline assembly sizes for both methods as well as the proportion of the transcriptome data that were mapped to the respective germline assemblies. Because of its superior performance, only the single-cell WGA assembly was used for further analyses; basic statistics and comparisons are provided in Table S1. Statistical analyses comparing different criteria of the different germline DNA isolation approaches were performed using R (v3.2.3) (49) and custom python scripts (http://github.com/maurerax/KatzLab/tree/SingleCellGermSoma).

### Gene family identification.

We used OrthoMCL (v5.0) (23) for identification of gene families from the core *C. uncinata* transcriptome, using default parameters (minimum similarity, 50%; minimum E value, 1E−5). This involved an initial all-versus-all BLAST analysis followed by MCL clustering, which ultimately provided a set of gene families and a list of their members (Table S3). Using custom python scripts, germline mapped members of gene families were sorted into bins for different categories (scrambled and nonscrambled).

### Estimation of gene family enrichment.

To test the distribution of scrambled transcript contributions to gene family sizes, we calculated the expected frequency of scrambled members based on the overall proportion of gene scrambling in the *Chilodonella* germline genome. We used these values to estimate the expected proportions of gene scrambling in each multimember gene family, and we used a a chi-square test to compare the observed and expected proportions of gene family members that were scrambled. The life cycle stage (found in the updated transcript names, see “Preparation of single-cell transcriptome data”) were used to identify the potential enrichment of a given life history stage in a particular gene family.

### Accession number(s).

Reads for both the genome and transcriptome assemblies were deposited in GenBank’s Short Read Archive (SRA) under BioProject number PRJNA413041.
