# Supplementary material for: Exploration of the Germline Genome of the Ciliate Chilodonella uncinata through Single-Cell Omics (Transcriptomics and Genomics)
Source: mBio. 2018 Jan 9;9(1):e01836-17. doi: 10.1128/mBio.01836-17 (PMC5760741; doi:10.1128/mBio.01836-17)
Supplement: TABLE S1 [file mbo001183657st1.docx]

Table S1. Comparisons of germline genome assemblies based on germline DNA gel-isolation method and single-cell techniques demonstrates superiority of single-cell WGA. Putative germline scaffolds are those with predicted ORFs across < 20% of their length while supported germline scaffolds had at least 3 transcripts that aligned to the scaffold. Somatic contamination (e.g. presence of telomere-containing scaffolds) are far more common and problematic in assemblies of Gel-isolated germline DNA. Similarly, using BLAST to map transcripts to the independent assemblies further demonstrates the superiority of the single-cell approach.

|  | **Gel-isolated DNA** | **Single-Cell WGA** |
| --- | --- | --- |
| **Number of reads** | 136,790,808 | 246,944,949 |
| **Number of Scaffolds** | 49,551 | 24,881 |
| **Putative Germline Scaffolds** | 420 | 2,751 |
| **Supported Germline Scaffolds** | 26 | 1,022 |
| **Scaffolds with Telomeres** | 9,222 | 57 |
| **Mapped Transcripts** | 468 | 5,019 |
| **Average Scaffold Length** | 195,422 | 25,975 |
