# Supplementary material for: Exploration of the Germline Genome of the Ciliate Chilodonella uncinata through Single-Cell Omics (Transcriptomics and Genomics)
Source: mBio. 2018 Jan 9;9(1):e01836-17. doi: 10.1128/mBio.01836-17 (PMC5760741; doi:10.1128/mBio.01836-17)
Supplement: TABLE S2 [file mbo001183657st2.docx]

Table S2. Top BLAST-hits for largest 250 regions of germline scaffolds without mapped transcriptome data that are significant above or below the average GC content. The majority of these atypically GC rich regions from the *C. uncinata* germline genome had homologs in other eukaryote taxa, predominantly other ciliate taxa and alveolates.

| **Eukaryote Hit** | **Germline Regions**  **2 S.D. Above Mean** |
| --- | --- |
| ***Tetrahymena thermophila*** | 43 |
| ***Paramecium tetraurelia*** | 36 |
| ***Stylonychia lemnae*** | 17 |
| ***Oxytricha trifallax*** | 11 |
| **Apicomplexa** | 4 |
| **Stramenopila** | 4 |
| **Other** | 21 |
