# Supplementary material for: Exploration of the Germline Genome of the Ciliate Chilodonella uncinata through Single-Cell Omics (Transcriptomics and Genomics)
Source: mBio. 2018 Jan 9;9(1):e01836-17. doi: 10.1128/mBio.01836-17 (PMC5760741; doi:10.1128/mBio.01836-17)
Supplement: TABLE S5 [file mbo001183657st5.docx]

Table S5. PCR primers used to discriminate between macro- and micronuclear copies of Actin.

| Primer Name | Target Genome | Sequence (5’ – 3’) |
| --- | --- | --- |
| Blue_MAC_Actin_53F | Soma | GGTACCGGTATGATCAAGGC |
| Actin_1080Rext | Soma | GTGATCCACATYTGYTGRAANGT |
| Blue_MIC_Actin_164F | Germline | GTACCATTGTCGATGACCACAG |
| Blue_MIC_Actin_913R | Germline | TTCCAGATCTTCTCCATGTAGTC |
