# Supplementary figures and images for: Exploration of the Germline Genome of the Ciliate Chilodonella uncinata through Single-Cell Omics (Transcriptomics and Genomics)
Source: mBio. 2018 Jan 9;9(1):e01836-17. doi: 10.1128/mBio.01836-17 (PMC5760741; doi:10.1128/mBio.01836-17)

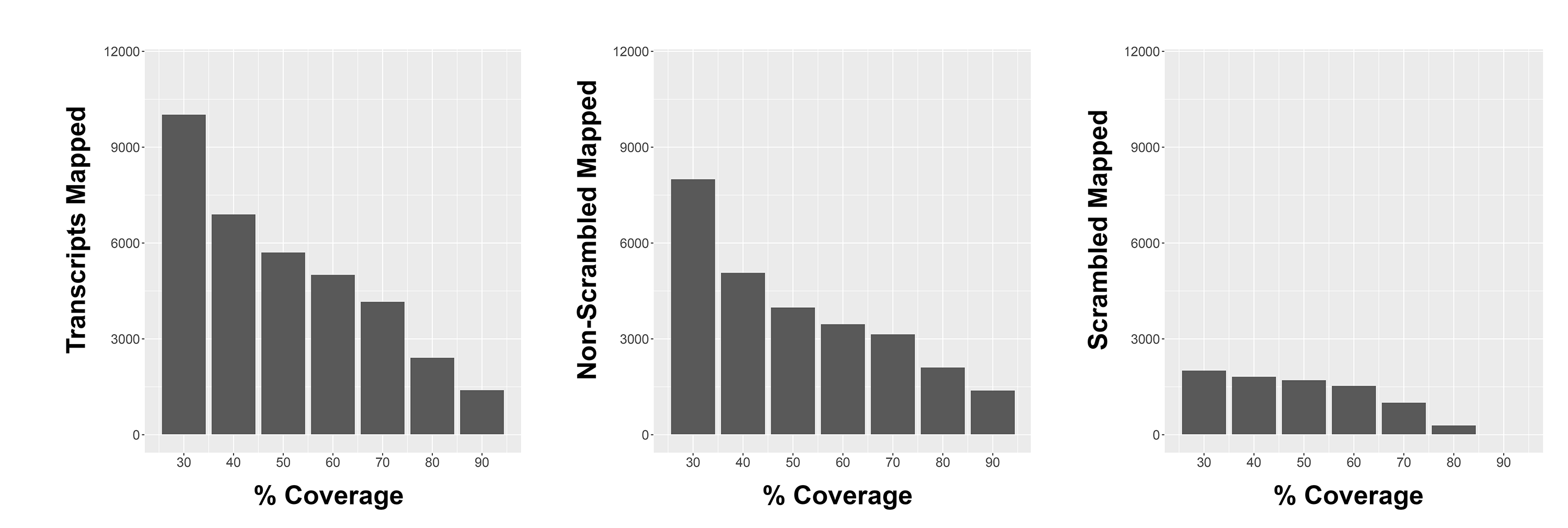

Supplement: FIG S1 [file mbo001183657sf1.tif]
